# Differential effects of palmitoleic acid on human lymphocyte proliferation and function

**DOI:** 10.1186/s12944-016-0385-2

**Published:** 2016-12-13

**Authors:** M. E. P. Passos, H. H. O. Alves, C. M. Momesso, F. G. Faria, G. Murata, M. F. Cury-Boaventura, E. Hatanaka, S. Massao-Hirabara, R. Gorjão

**Affiliations:** 1 Institute of Physical Activity and Sport Sciences, Interdisciplinary Post-graduate Program in Health Sciences, Cruzeiro do Sul University, Rua Galvão Bueno, 868, Liberdade, CEP: 01506 000 São Paulo, SP Brazil; 2 Department of Physiology and Biophysics, Institute of Biomedical Sciences, University of São Paulo, São Paulo, Brazil

**Keywords:** Th1 cells, T regulatory cells, Fatty acids, Cell proliferation

## Abstract

**Background:**

Palmitoleic acid (PA) is a n-7 monounsaturated fatty acid (MUFA) secreted by adipose tissue and related to decreased insulin resistance in peripheral tissues. Evidences have been shown that PA also decreased proinflammatory cytokine expression in cultured macrophages. Although studies have shown that other fatty acids (FAs) modulate several lymphocyte functions, the specific effect of PA on these cells is unknown. The aim of the present study was to evaluate the possible influence of PA on activation and differentiation of human lymphocytes in comparison to oleic acid (OA).

**Methods:**

Human lymphocytes were isolated from peripheral blood of health men and cultured in the presence of growing concentrations of PA or OA (5 to 200 μM), for 24 h. After that, cells were collected and cytotoxicity evaluated by flow cytometry. Then, we analyzed proliferative capacity in lymphocytes treated with non toxic concentrations of PA and OA (25 and 50 μM, respectively), in the presence or absence of concanavalin A (ConA). The Th1/Th2/Th17 cytokine production was determined by the *Cytometric Bead Array*. CD28 and CD95 surface expression and T regulatory cell percentage were determined by flow cytometry.

**Results:**

We observed that PA is toxic to lymphocytes above 50 μM. PA promoted a decrease of lymphocyte proliferation stimulated by ConA in both concentrations. PA also decreased CD28 externalization and increased CD95. On the other hand, OA did not alter these parameters. In the same way, PA reduced IL6, IFN-gamma, TNF-alpha and IL17A production in both concentration and IL2 only at 50 μM (in the presence of ConA). OA promoted IFN-gamma reduction in both concentrations and an increase of IL-2, IL4 and IL10 at 25 μM. Both fatty acids decreased the percentage of T regulatory cells.

**Conclusion:**

In conclusion, PA promoted a suppressive effect on lymphocyte proliferation characterized by a decrease of Th1 and Th17 response, and co-stimulatory molecule (CD28). However, OA increased lymphocyte proliferation through IL2 production and Th2 response. These results also show a more suppressive effect of PA on lymphocytes in comparison to OA.

## Background

Currently, it is well known that fatty acids (FAs) modulate leukocyte function. Monounsaturated FAs are considered less toxic to lymphocytes when compared to polyunsaturated fatty acids [1]. The influence of FAs in the control of inflammatory processes has been studied due to the ability of these compounds to be incorporated into the cell membranes, resulting in the production of eicosanoids with lower inflammatory effects [2, 3]. FAs also can bind in membrane receptors or change intracellular protein activation generating alterations in cellular function. Some fatty acids modulate the toll like receptor-4 induced signaling pathways promoting alterations in inflammatory process [4] Studies have shown that monounsaturated FAs modulate important transcription factors involved with inflammatory pathways such as NF-kB [5]. The different types of FAs in the diet can modulate the lymphocyte proliferative capacity and cytokine production. Th1 cells are more sensitive to the effects of FAs when compared to Th2 lymphocytes [6].

The monounsaturated FAs have different effects on the organism, as demonstrated by studies that replaced saturated FAs by monounsaturated FAs of the diet [7, 8]. Previous studies of our group showed that the oleic acid (OA) stimulates cell proliferation in low concentrations (12.5 and 25 μM) and decreases the proliferation in higher concentrations (above 75 μM) [9]. In contrast, supplementation with olive oil in vivo, which is rich in OA, promotes a decrease of lymphocyte proliferation stimulated with Concanavalin A (ConA) [10]. Linos et al. [11] also observed that the supplementation with olive oil presents a beneficial effect on the exacerbated inflammatory response in patients with rheumatoid arthritis, due to an immunosuppressive effect.

Macadamia oil presents a higher proportion of palmitoleic acid (PA) when compared to other oils. Supplementation with this oil promotes a decrease of serum triglyceride and cholesterol levels, as well as it can be related to the reduced risk of cardiovascular diseases development [12]. Other studies have shown that macadamia oil normalizes levels of HDL and LDL-cholesterol in individuals with hypercholesterolemia [13, 14].

In addition to the diet, PA also is produced and released by adipose tissue [15]. This FA is synthesized in adipose tissue by the stearoyl-coenzyme A (SCD), an enzyme that desaturates palmitic acid (16: 0) to palmitoleic acid (16:1) [16]. It has been demonstrated that PA is related to the increased insulin sensitivity in the liver and muscle [15], and improved hyperglycemia and hypertriglyceridemia through increasing insulin sensitivity and altering liver lipid metabolism in diabetic rats [17, 18]. Bolsoni et al. [19] demonstrated that PA also increased lipolysis and decreased the lipogenesis in adipocytes. Evidences have been shown that PA also decreased NF-kB p65 phosphorylation and proinflammatory cytokine expression in cultured macrophages [5]. These results are indicative of possible anti-inflammatory effects promoted by PA. Plasma PA concentration can be altered in other physiological conditions. Tepisc et al. [20] observed an increase of PA in plasma of professional football and basketball players compared to sedentary human of the same age, but the consequences of these differences are not known.

The studies about PA effects have been increased in the last years, however there are few studies showing the influence of PA on immune system, specifically in leukocytes. Since these cells are in communication with multiple tissues, including adipose tissue [21], any changes in the levels of PA can modulate their function. Therefore, the aim of the present study was to evaluate the effects of PA on activation and differentiation of human lymphocytes in comparison to OA.

## Methods

### Isolation of peripheral blood lymphocytes

Human lymphocytes were obtained from the peripheral blood of males (20–45 years) with no history of chronic inflammatory or autoimmune diseases, infections or, other chronic diseases (*diabetes* and dyslipidemias). Individuals were instructed to not perform any physical activity for the last 24 h before blood collection. All volunteers have signed a consent form. This study was approved by Cruzeiro do Sul University Ethics Committee in Human Research (Protocol CE/UCS-084/2012).

Peripheral blood lymphocytes were obtained as described by Böyum et al. [22]. Blood was layered on Histopaque ® 1077 reagent (Sigma Chemical Co, St. Louis, MO, EUA) and centrifuged, for 30 min at 400 *g*, at room temperature. Mononuclear cells, collected from the interphase, were incubated for 1 h at 37 °C and 95% atmospheric air 5% CO_2_, under sterile conditions into a 75 cm^2^ culture bottle, containing RPMI-1640 culture medium, enriched with 2 mM glutamine, 24 mM sodium bicarbonate, 20 mM HEPES, 10% fetal bovine serum (FBS), and antibiotics (1000 U/mL penicillin and 1000 μg/mL streptomycin). Monocytes adhered to the bottle were discarded. Lymphocytes, remained in the supernatant, were isolated by centrifuging at 400 *g* for 10 min. The cells were resuspended in phosphate-buffered saline (PBS).

### Lymphocyte culture and treatments

Lymphocytes were cultured at 1 × 10^6^ cells per mL in all experiments. The toxic concentration of PA in primary culture of human lymphocytes was determined previously to the function and cellular activation experiments. Initially, cells were incubated with concentrations from 25 to 200 μM of PA for 24 h. The toxic concentration of OA (50 μM) to human lymphocytes was determined previously by Cury-Boaventura et al. [1]. Cells from control group were treated with ethanol with a concentration of 0.05% in all experiments, which one does not have toxic effects to human lymphocytes [23].

### Determination of membrane integrity and externalization of phosphatidylserine

Externalization of phosphatidylserine and membrane integrity were analyzed by flow cytometry using the *FITC Annexin V/Dead Cell Apoptosis Kit* (Invitrogen, Paisley, UK) according to the method described by Vermes et al. [24].

Cells were cultured in RPMI 1640 medium under the same conditions described above. After 24 h of FA treatments, lymphocytes were resuspended in PBS. Posteriorly, 500 μL of each sample were transferred to conical tubes. The suspension was centrifuged at 400 *g* for 10 min and resuspended in 100 μL of annexin buffer (Binding Buffer: 10 mM HEPES/NaOH, 140 mM NaCl and 2.5 mM CaCl_2_). 5 μL of fluorescein-conjugated annexin V (annexin V-FITC 20 μg/mL in 25 mM HEPES - 140 mM NaCl, 1 mM EDTA, pH 7.4, and 0.1% bovine serum albumin) were added and the cells incubated for 15 min in the dark, at room temperature. Afterwards, 1 μL of propidium iodide (PI) (100 μg/mL) and 400 μL of buffer were added to these samples and analyzed by flow cytometry (FACS Aria II, Becton Dickinson, CA, USA). Ten thousand events per sample were acquired using filters for PI and FITC fluorescence. The histograms were then analyzed using the *BD-Diva software* (Becton Dickinson).

### DNA fragmentation in lymphocytes

DNA fragmentation was performed by flow cytometry, according to the method described by Nicoletti et al. [25]. Cells (5 × 10^5^) were resuspended in 500 μL of a hypotonic solution containing PI (50 μg/mL, 0.1% sodium citrate, and 0.1% Triton X-100). Cells were then incubated for 30 min, at 25 °C, and the fluorescence measured as described above.

### Cell proliferation assay

Cell proliferation was evaluated as described by Gorjao et al. [9]. Briefly, lymphocytes were isolated and resuspended in 1 mL of RPMI-1640 medium, supplemented as described above. Afterwards, 2.5 × 10^5^ lymphocytes per well were cultured in 96-well microtiter plates, in 200 μL of RPMI medium, containing non-toxic concentrations of 25 μM and 50 μM of PA or OA. Treatments with the FAs were performed in the presence or absence of Concanavalin A (5 μg/mL) (Sigma Chemical Co.). After the period of 30 h, [2-^14^C] thymidine (1 μCi per mL) was added to the culture medium and cells were incubated for an additional 18 h period. At the end, cells were automatically collected using the *Multiple Skatron Combi Cell Harvester* (Sulfolk, UK). The counting of incorporated radioactivity was performed using the *Beckman-LS 5000TD Counter* (Beckman Instruments, Fullerton, CA, USA).

### Analysis of CD28 and CD95 Expression on the Lymphocyte Surface and of percentage of T regulatory lymphocytes

After the FA treatments, in the presence and absence of ConA for 24 h, the expression of CD95 and CD28 on the lymphocyte surface was performed by flow cytometry. The cell suspension (1 × 10^6^ cells) was centrifuged at 400 *g* for 10 min, followed by washing twice with PBS containing 1% albumin. Specific antibodies conjugated to PerCP-Cy5 (CD28) or APC (CD 95) were added to the cell suspensions (1:20), which ones were incubated at room temperature for 30 min, in the dark. Negative control cells were incubated with labeled IgG antibody. After this period, cells were washed twice with PBS and analyzed on the flow cytometer: FACS CD28 and CD95 in Aria II (Becton Dickinson, CA, USA) and BD Accuri the CD25 (Becton Dickinson). Histograms were analyzed using the *BD Diva Software* or *BD C6 Sampler Accuri Software* by determining the fluorescence through the specific filters for each fluorochrome.

Determination of percentage of regulatory T cells in total lymphocyte culture after the FA treatments, in the presence or absence of ConA for 48 h, was performed by flow cytometry. Cells (1 × 10^6^ cells) were centrifuged at 400 *g,* for 15 min, followed by washing twice with PBS containing 1% albumin (BSA) and resuspended in 100 μL of same buffer. The determination of Treg cell percentage was performed using *Foxp3* ^*+*^ *Human Kit* (Becton Dickinson, CA, USA), according to manufacturer’s instructions. Briefly, the specific antibodies anti-CD4 (FITC) and anti-CD25 (APC) were added to the suspension of lymphocytes (1:20) and the cells incubated at room temperature, for 30 min, in the dark. Negative control cells were incubated with the non-reactive labeled IgG antibody. After this period, cells were fixed (1% formaldehyde in PBS) and incubated in buffer containing a permeabilizing agent (Becton Dickinson), for 15 min. Subsequently, cells were washed with PBS containing 1% BSA, followed by incubation with anti-Foxp3 (PE) antibody, during 30 min (1:20 dilution). Twenty thousand events per sample were acquired by the flow cytometer BD-Accuri. Firstly, determination of CD4^+^ cells was performed and, from these cells, the percentage of CD25^+^/Foxp3^+^ cells was determined. Histograms were then analyzed using the *BD Accuri C6 Software*.

### Determination of cytokine production in cultured lymphocytes: Th1, Th2 and Th17 profile

After the FA treatments, in the presence and absence of ConA for 24 h, the measurement of TNF-α, IL-6, IL-4, IL-2, IL-10, IFN-γ, and IL-17A concentrations in the lymphocyte culture supernatant was performed using the *BD™ Cytometric Bead Array (CBA) Human Th1/Th2/Th17 Cytokine kit* (BD Biosciences), according to manufacturer’s instructions. These cytokines are largely produced by Th1, Th2, and Th17 lymphocytes.

Briefly, 25 μL of particles containing different fluorescent beads and covered with specific antibodies for the cytokines were added to 25 μL of diluted culture supernatant and incubated for 1 h, at room temperature in the dark. Afterwards, 25 μL of the secondary antibody conjugated to a fluorochrome were added to the suspension, followed by the incubated for 2 h, at room temperature. At the same time, the standards for each cytokine were similarly used in the absence of the samples. The particles were washed to remove the unbound antibodies, resuspended in washing buffer and analyzed by using the *BD Accuri* (BD Biosciences). The acquisition was made in *BD-Accuri C6 Software* and the cytokine concentrations determined using the *FCAP Software v.3.0* (BD, Biosciences).

### Statistical analysis

For statistical analysis of toxicity tests, the *One-way ANOVA* test followed by Tukey’s post-test was applied, considering significant differences when *p* < 0.05. For statistical analysis of the experiments performed in the presence or absence of ConA, the *Two-way ANOVA* test followed by Bonferroni post-test was performed, considering significant differences when *p* < 0.05. Statistical analysis was performed using *GraphPad Prism 5* (GraphPad Software, USA).

## Results

### Cell viability assay

**Fig. 1:**
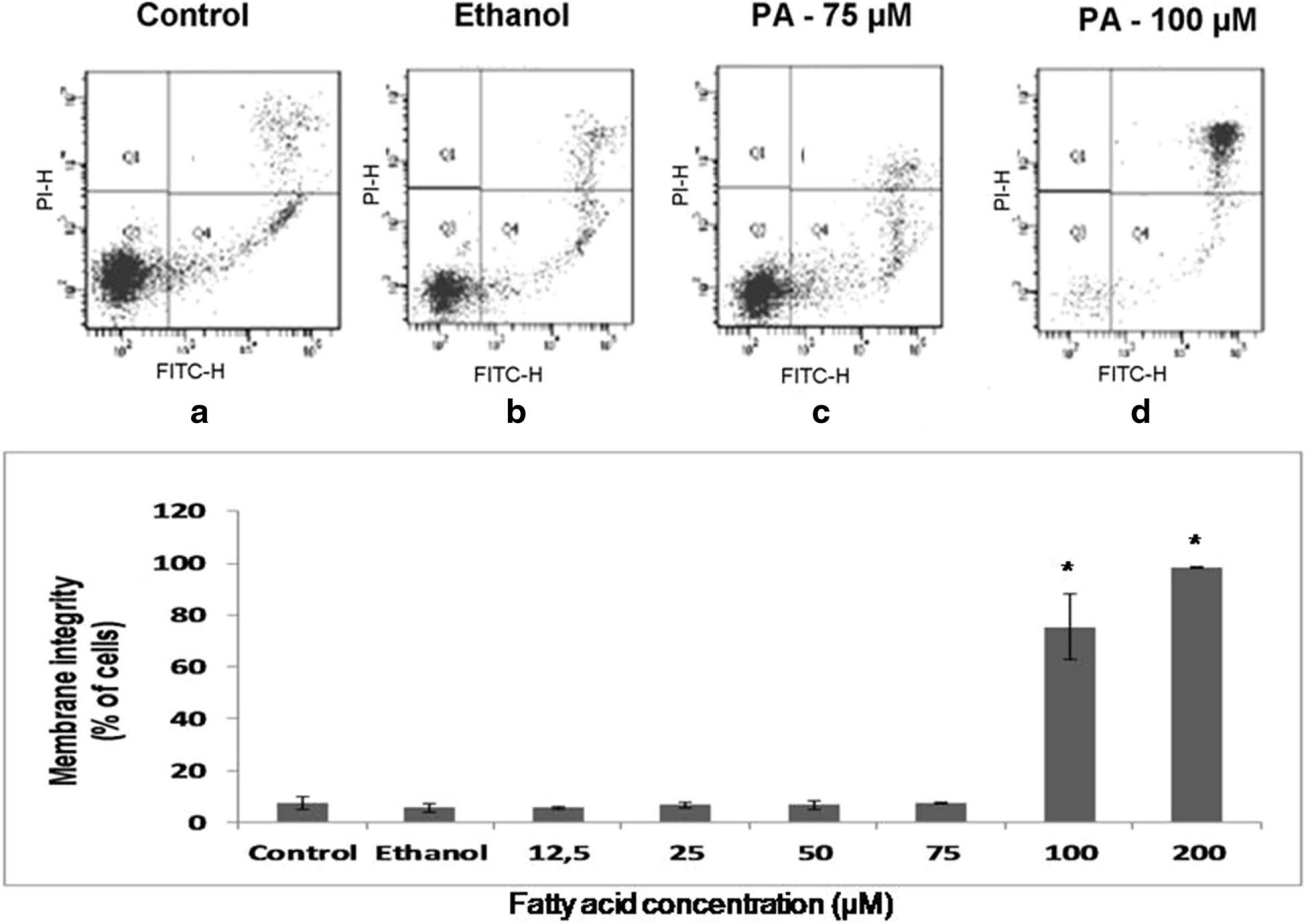
Effect of palmitoleic acid on membrane integrity and phosphatidylserine externalization of lymphocytes. After treatment with 12.5 to 100 μM of PA for 24 h, lymphocytes were incubated with propidium iodide and Annexin V-FITC and analyzed by flow cytometry. Representative dot plots related to PI (*y axis*) and FITC (*x axis*) fluorescence are shown in: **a** Control cells incubated only with RPMI medium; **b** Cells treated with 0.05% of ethanol; **c** Cells treated with 75 μM of PA; **d** Cells treated with 100 μM of PA. The values are presented in the graph below as mean ± S.E.M. of three determinations from six experiments. *P < 0.01 versus control or ethanol

**Fig. 2:**
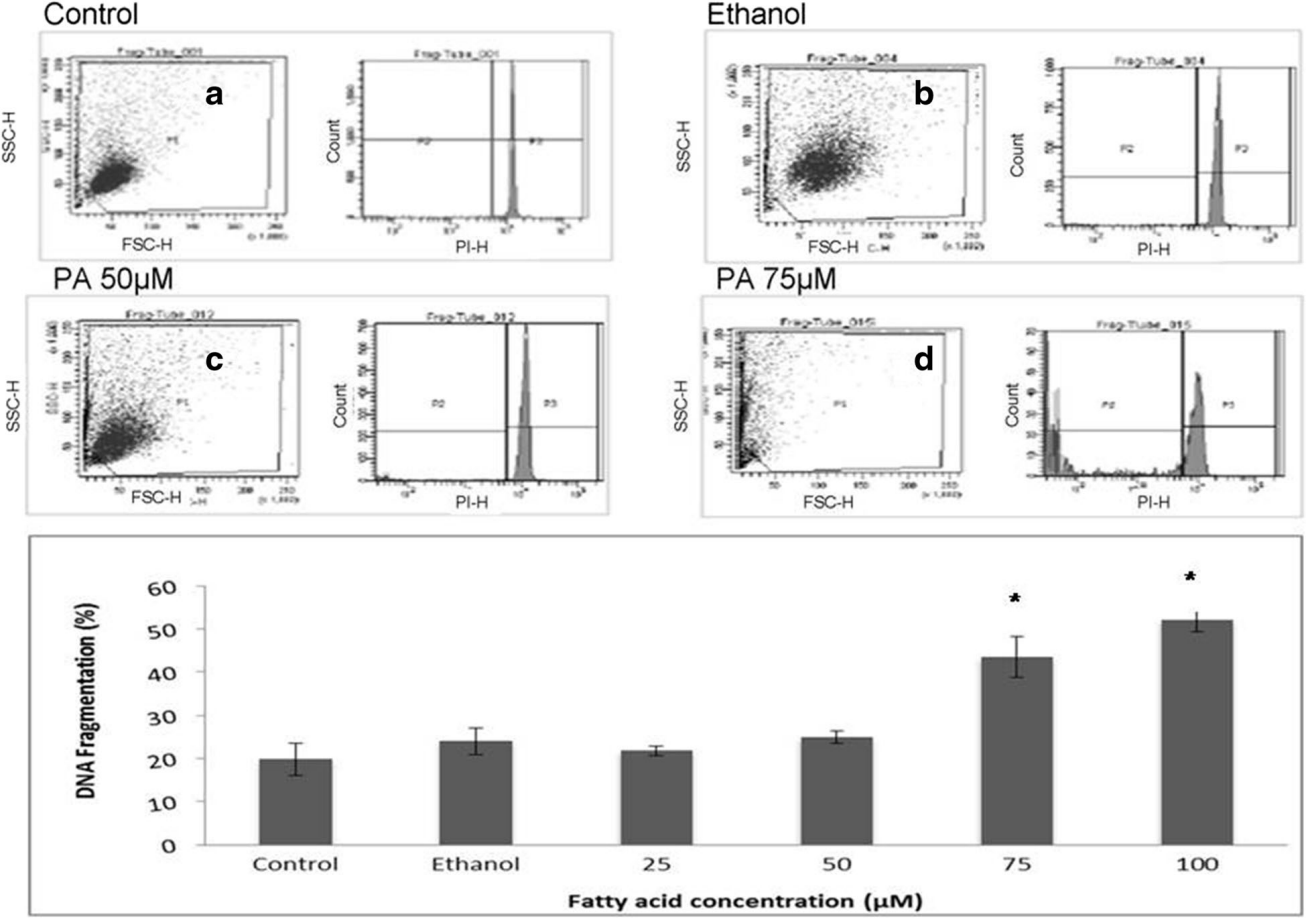
Effect of palmitoleic acid on human lymphocyte DNA fragmentation. After treatment with 12.5 to 100 μM of PA for 24 h, cells were stained with a buffer containing citrate, Triton X100 and propidium iodite and analyzed by flow cytometry. The percentages correspond to the fraction of cells with fragmented DNA. Representative dot plots of the forward-light scatter (*x axis*) and side-light scatter (*y axis*) used as indicators of cell size and granularity respectively, and histograms from PI fluorescence are shown in: **a** Control cells incubated only with RPMI medium; **b** Cells treated with 0.05% of ethanol; **c** Cells treated with 50 μM of PA; **d** Cells treated with 75 μM of PA. The values are presented in the graph below as mean ± S.E.M. of three determinations from six experiments. *P < 0.01 versus control or ethanol

In the evaluation of membrane integrity and phosphatidylserine externalization assays, PA promoted loss of 72% of membrane integrity in lymphocytes treated during 24 h at 100 μM (Fig. 1). For analysis of lymphocyte DNA fragmentation (Fig. 2), we observed that PA induced an increase of 20% of DNA fragmentation in concentrations higher than 50 μM, when compared to control and ethanol. These results are indicative that PA is toxic to lymphocytes in concentration above 50 μM.

### Evaluation of lymphocyte proliferative capacity

**Fig. 3:**
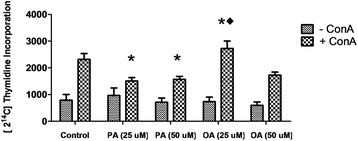
Effect of palmitoleic acid (PA) and oleic acid (OA) at non-toxic concentration on Concanavalin A induced human lymphocyte proliferation. After treatment with 25 and 50 μM of PA or OA in the presence or absence of ConA for 30 h, cells were incubated with 2 [^14^C]-thymidine (1 μCi/mL). Afterwards, cells were collected and radioactivity counted. Data are expressed as counts per minute (cpm) and presented as mean ± S.E.M. of six determinations from six experiments. *P < 0.05 versus control. ♦*P* < 0.05 versus 25 μM of PA

Evaluation of PA effect on lymphocyte proliferation in non-toxic concentration is important to identify possible immunosuppressive effects of this fatty acid. In relation to this parameter, PA decreased around 50% the ConA-stimulated lymphocyte proliferation in both concentrations evaluated (25 and 50 μM). On the other hand, the treatment with OA increased 17% lymphocyte proliferative capacity at the concentration of 25 μM, in the presence of ConA (Fig. 3). These results are indicative that PA has immunosuppressive effects that are not related to its toxicity.

### Evaluation of CD28 and CD95 expression on lymphocyte surface

**Fig. 4:**
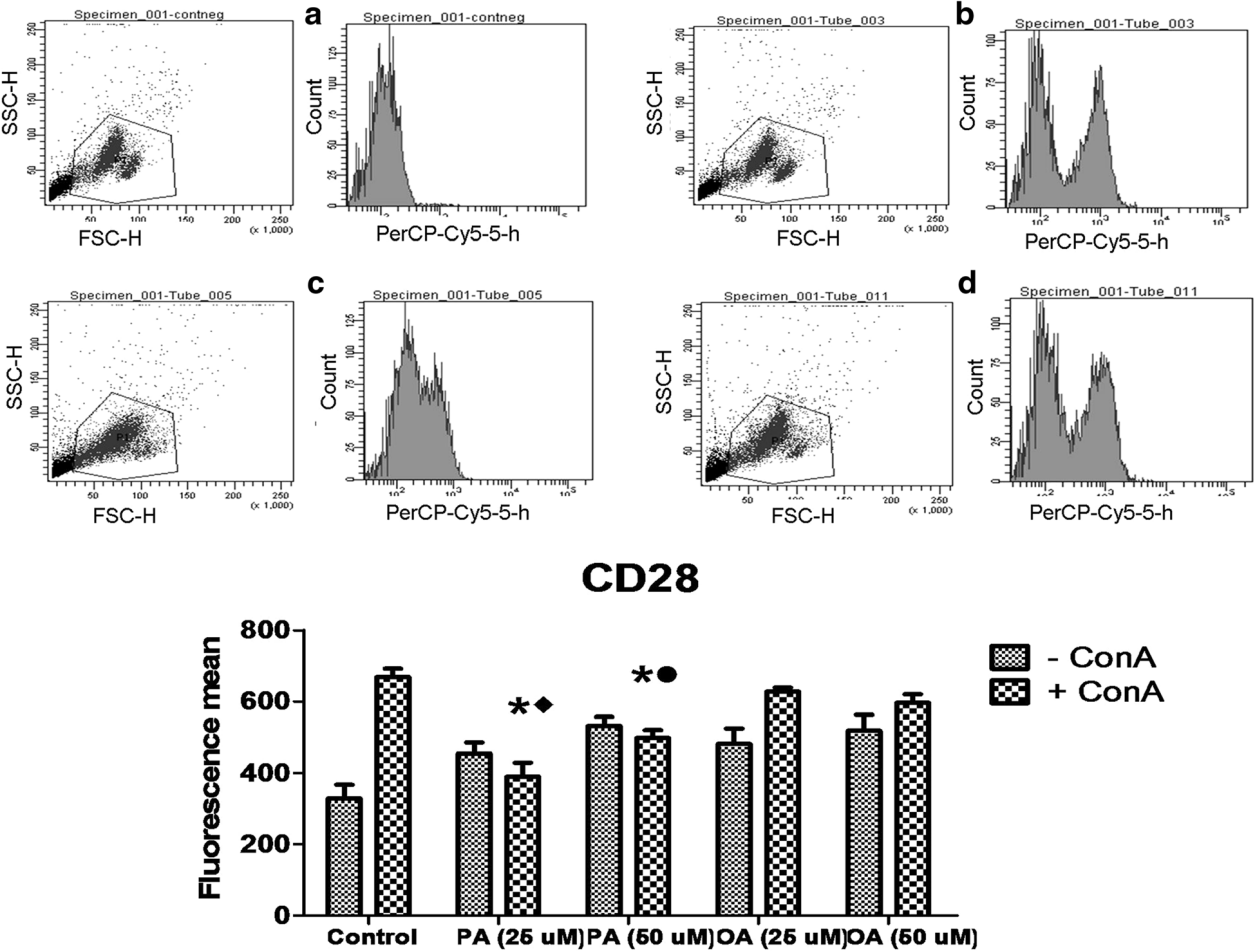
Effect of palmitoleic acid (PA) and oleic acid (OA) at non-toxic concentration on human lymphocyte CD28 membrane surface expression. Cells were incubated with 25 and 50 μM of PA or OA in the presence or absence of ConA for 24 h. After, cells were stained with anti- PerCP-Cy5- CD28 and analyzed by flow cytometry. Negative controls were incubated with non-reactive IgG. Representative dot plots of the forward-light scatter (*x axis*) and side-light scatter (*y axis*) used as indicators of cell size and granularity respectively, and histograms from PerCP-Cy5 fluorescence (related to CD28 detection) are shown in: **a** Negative controls; **b** ConA stimulated control cells; **c** 25 μM PA + ConA treated cells; **d** 25 μM OA + ConA treated cells. The values are presented in the graph below as mean ± S.E.M. of three determinations from six experiments. *P < 0.01 versus control. ♦*P* < 0.05 versus 25 μM of OA. •*P* < 0.05 versus 50 μM of OA

Determination of molecules related to lymphocyte activation or suppression is important to evaluate the impact of PA on these cells. OA treatment did not induce any change in the CD28 expression. The treatment with PA promoted a decrease of 41.2 and 19% in the expression of CD28 at both concentrations evaluated (25 and 50 μM, respectively) when compared to the control cells and a decrease of 36 and 9% when compared to cells treated with 25 and 50 μM of OA (Fig. 4).

**Fig. 5:**
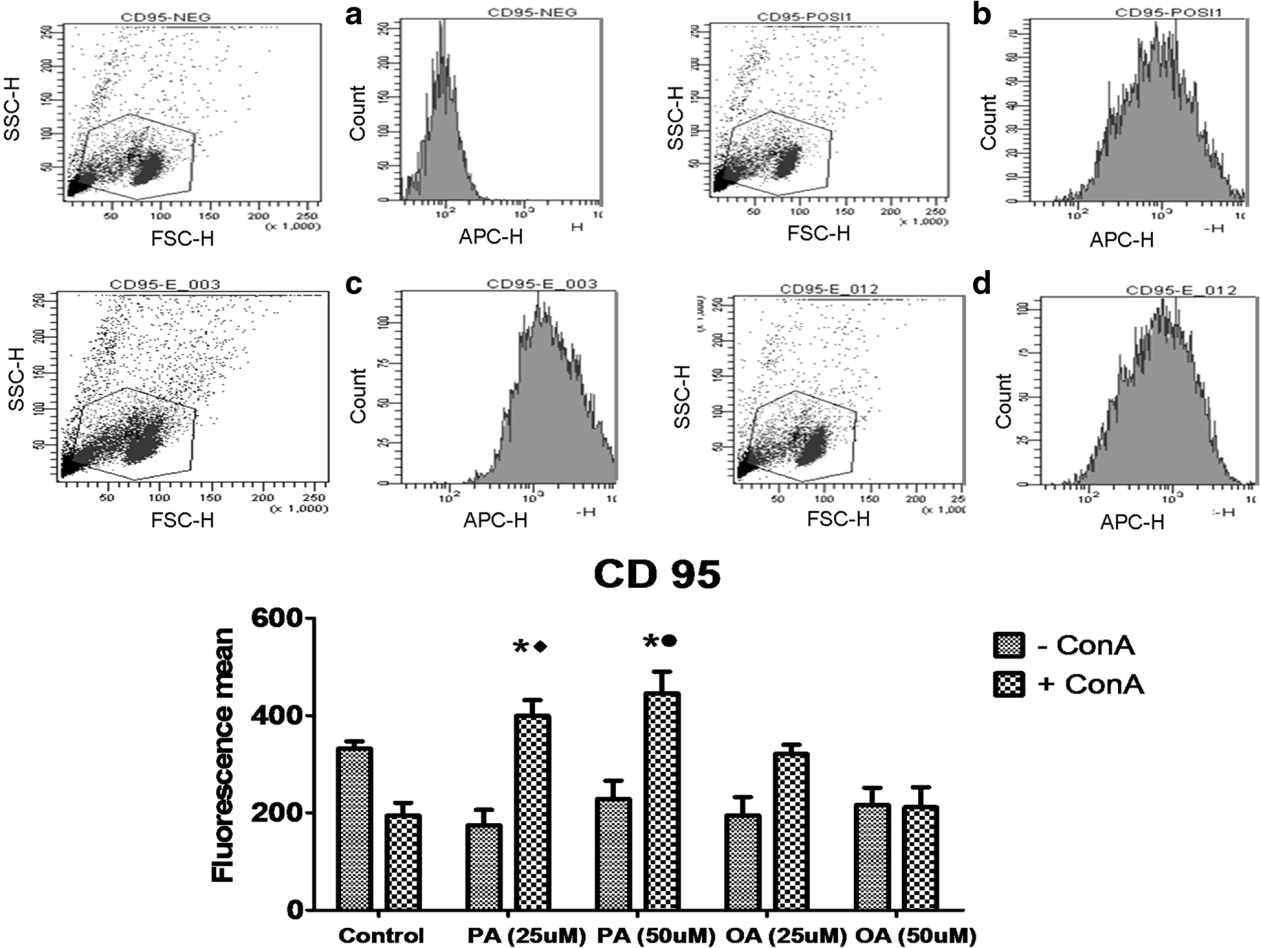
Effect of palmitoleic acid (PA) and oleic acid (OA) at non-toxic concentration on CD95 human lymphocyte membrane surface expression in. Cells were incubated with 25 and 50 μM of PA or OA in the presence or absence of ConA for 24 h. After, cells were stained with anti-APC-CD95 and analyzed by flow cytometry. Negative controls were incubated with non-reactive IgG. Representative dot plots of the forward-light scatter (*x axis*) and side-light scatter (*y axis*) used as indicators of cell size and granularity respectively, and histograms from APC fluorescence (related to CD95 detection) are shown in: **a** Negative controls; **b** ConA stimulated control cells; **c** 25 μM PA + ConA treated cells; **d** 25 μM OA + ConA treated cells. The values are presented in the graph below as mean ± S.E.M. of three determinations from six experiments. *P < 0.01 versus control. ♦*P* < 0.05 versus 25 μM of OA. •*P* < 0.05 versus 50 μM of OA

Cells treated with PA increased 105 and 130% the expression of CD95 at 25 and 50 μM, respectively, in relation to control cells. CD95 expression in cells treated with PA was 24.2 and 111% higher in relation to OA treated cells with 25 and 50 μM. OA treatment did not affect CD95 expression in lymphocytes (Fig. 5).

### Evaluation of Treg cell percentage of (CD4^+^, CD25^+^, Foxp3^+^)

**Fig. 6:**
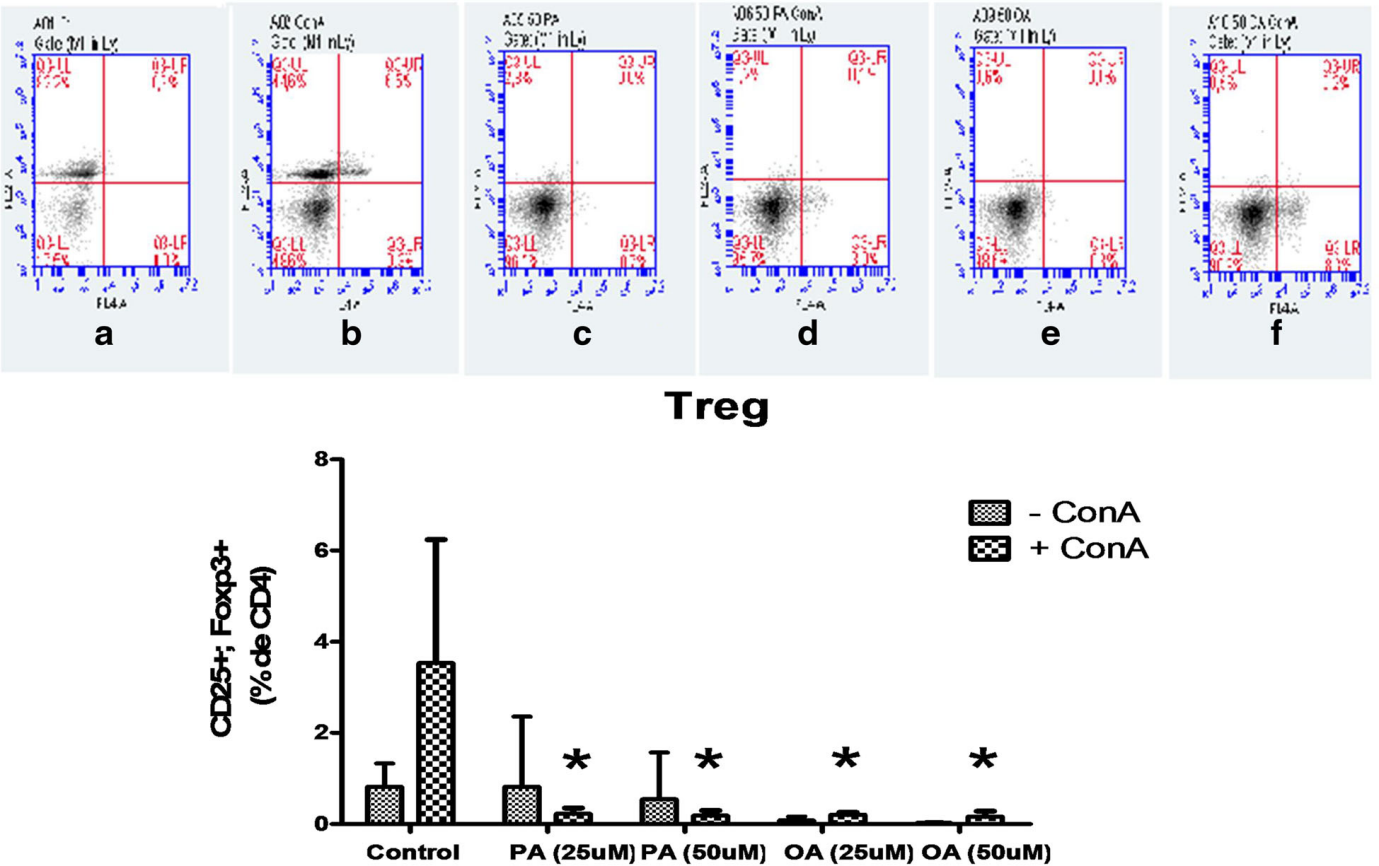
Effect of palmitoleic acid (PA) and oleic acid (OA) at non-toxic concentration on T regulatory cell differentiation. Cells were incubated with 25 and 50 μM of PA or OA in the presence or absence of ConA for 24 h. After, cells were stained with anti-CD4 (FITC) and anti-CD25 (APC). The negative control cells were incubated with non-reactive labeled antibody IgG. After this period, the cells were fixed and incubated in buffer containing a permeabilizing agent followed by incubation with anti-Foxp3 (PE). Twenty thousand events in CD4+ cells per sample were acquired in histograms on the flow cytometer BD-Accuri. At first, a determination of CD4+ cells was performed and from these, the percentage of CD25 + Foxp3 + cells was determined. Representative dot plots of APC fluorescence (*x axis*) and PE fluorescence (*y axis*) used as indicators of CD25 and Foxp3 are shown in: **a** Control cells; **b** ConA stimulated control cells; **c** 50 μM PA treated cells; **d** 50 μM PA + ConA treated cells; **e** 50 μM OA treated cells; **f** 50 μM OA + ConA treated cells The values are presented as mean ± S.E.M. of three determinations from six experiments. *P < 0.01 versus control

Determination of Treg cell differentiation is important due to the inhibitory effect of these cells on lymphocyte proliferation control. Treatment with PA reduced the amount of Treg cells from 3.53 to 0.22 and to 0.19% of CD4+ cells when compared to the control cells at 25 and 50 μM, respectively (Fig. 6). Surprisingly, OA also decreased the percentage of Treg cells from 3.53 to 0.20 and to 0.17% of CD4+ cells at 25 and 50 μM respectively, when compared to the control cells. This results are indicative that both FAs evaluated can modulate Treg cell differentiation. However, the final effect of these two FAs is different as observed in proliferation data.

### Evaluation of cytokine concentrations in cell supernatant

Th1, Th2 and Th17 cytokine secretion by stimulated lymphocytes treated with non-toxic PA or OA concentration was evaluated.

**Fig. 7:**
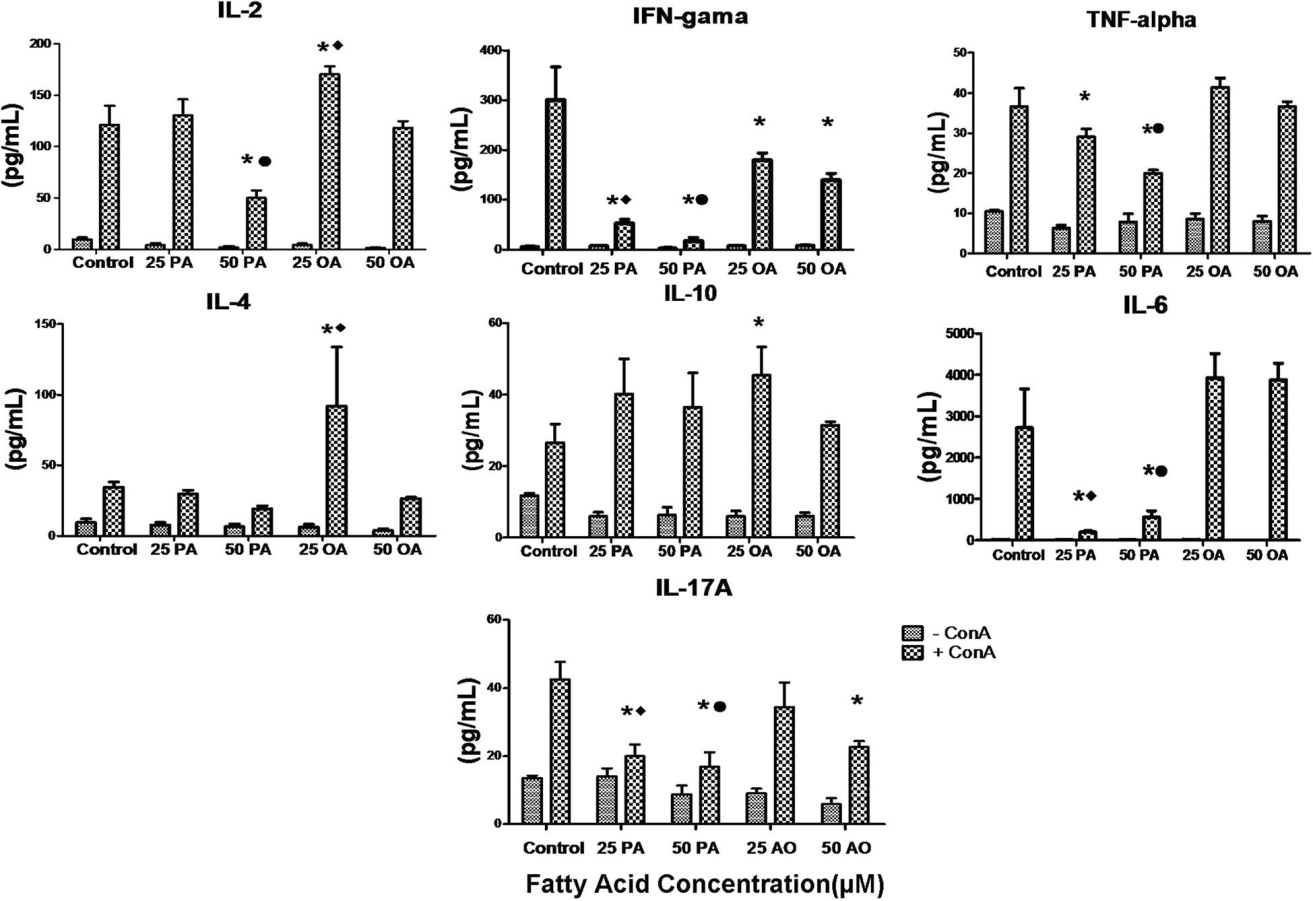
Effect of palmitoleic acid (PA) and oleic acid (OA) at non-toxic concentration on human lymphocyte cytokine production. Cells were incubated with 25 and 50 μM of PA or OA in the presence or absence of ConA for 24 h. After, supernatant was collected and IL-2, IFN-gamma, TNF-alpha, IL-4, IL-10, IL-6 and IL-17 concentration determined by Cytometric Bead Array. The values are presented as mean ± S.E.M. of three determinations from six experiments. *P < 0.01 versus control. ♦*P* < 0.05 versus 25 μM of OA. •*P* < 0.05 versus 50 μM of OA

Cells treated with 50 μM of PA presented a reduction of around 64% in the production of IL-2 compared to the cells treated with 50 μM of OA and to the control cells, in the presence of ConA. OA increased 25 and 20% IL-2 production at 25 μM when compared to the control and PA (25 μM) groups, respectively (Fig. 7). Cells treated with PA in both concentrations (25 and 50 μM) presented a reduction in the production of IL-6 (90 and 81.8% respectively) when compared to the control group. In the comparison to OA group, PA treated lymphocytes presented 93.6 and 87.1% lower secretion of this cytokine at 25 and 50 μM, respectively. We also observed a reduction of 83.3 and 93.3% in the production of IFN-γ when the cells were treated with PA at 25 and 50 μM when compared to control group (Fig. 7). The treatment with OA also promoted a decrease of 40 and 50% of IFN-γ secretion in relation to control group.

TNF-alpha production was reduced 23.6 and 47.4% by the treatment with PA at 25 and 50 μM respectively, in comparison to control cells. The decrease induced by the treatment with 50 μM of PA was 47% lower when compared to the treatment with 50 μM of OA (Fig. 4).

These data are suggestive that PA modulates inflammatory Th1 response leading to a suppression of cytokines produced by these cells.

IL-4 production was elevated in lymphocytes treated with 25 μM of OA when compared to the PA treatment and to the control cells (Fig. 7). PA did not alter production of IL-4. IL-10 concentration was increased in lymphocytes treated with 25 μM of OA in comparison to the control cells. There were no differences between OA and PA groups at any concentration for this cytokine (Fig. 7). These data show data the PA treatment did not affect Th2 response.

We also observed an IL-17A reduction in lymphocytes treated with PA compared to control (reduction of 55.5 and 60%) and OA groups, at both concentrations (25 and 50 μM). OA promoted a reduction of 33.3% of this cytokine only at 50 μM (Fig. 7). These results indicate that PA and OA modulate Th17 response.

## Discussion

Studies have shown that PA plasma concentration can be modulated in several conditions and it is related to important metabolic alterations. However, this is the first study to show direct PA effects on lymphocytes. Firstly, we analyzed the toxic concentration of PA on these cells. We found that PA promotes death of lymphocytes at concentration above 50 μM. The toxicity of PA was similar to OA, as showed in previous studies of our group [1, 9]. FAs, at high concentrations, can induce leukocyte death by apoptosis or necrosis, modifying the cell activation process. However, at low concentrations (25 μM), OA stimulates proliferative capacity in lymphocytes, showing the importance of evaluate FA effects at different non-toxic concentrations.

Therefore, we evaluated the PA effects in non-toxic concentrations (25 and 50 μM) on the proliferative capacity of lymphocytes. This FA promoted a reduction in cell proliferation compared to the control group, in the ConA-stimulated condition. These results suggest that PA has a suppressive effect on lymphocyte activation even at non-toxic concentrations, and that this effect is different to that observed by the OA treatment.

Lymphocytes activation is usually modulated by expression of co-stimulatory molecules on the cell membrane and by the action of several cytokines. Cytokines play a crucial role in controlling the expansion of T cells during immune response to pathogenic antigens. Cytokines also regulate the processo of differentiation of these cells, depending of the required response [26–29]. We determined the concentrations of several cytokines released by lymphocytes into the culture supernatant under different conditions. We found that PA reduced IL6, IFN-γ, TNF-α, and IL17A content at both concentrations evaluated, and IL-2 level only at 50 μM. These cytokines exhibit inflammatory characteristics, leading to lymphocyte activation, mainly addressing a Th1 and Th17 response. The effects of PA on cytokine production can be involved with the suppressive role of this FA on lymphocyte proliferation. We also observed that OA promoted a reduction of IFN-γ content at both concentrations and an increase of IL-2, IL-4, and IL-10 levels at 25 μM. The IL-2 cytokine is essential for stimulating cell proliferation [9] and it can be a possible pathway involved in the OA effect on the activation of cell proliferation at 25 μM. Additionally, IL-4 and IL-10 induced a Th2 response, which one would be specific for reactions related with hypersensitivity. Therefore, reduction in Th1 and Th17A cytokine release by the PA treatment was not associated with Th2 response.

In addition to the modulation of cytokine production by PA, this FA can influence peripheral activation of lymphocytes. We analyzed the percentage of Treg cells (CD4^+^/CD25^+^/Foxp3^+^), which one exhibits suppressive characteristics. We found that both PA and OA promoted a reduction in the percentage of these cells in both concentrations when compared to control cells. Treg lymphocytes reduce the clonal expansion of Th1 and Th17A cells and, consequently, decreasing the inflammatory response [30, 31]. However, in the present study, PA inhibited proliferative capacity of lymphocytes and the production of inflammatory cytokines (Th1 and Th17 response), as well as the percentage of Treg cells. These results suggest that the suppressor effect of PA on lymphocyte proliferation and activation may involve different regulatory mechanisms. On the other hand, the inhibitory effect of PA on IL-2 production can be related to Treg percentage decrease, since this cytokine has been associated to Treg cell differentiation [32].

Among the possible mechanisms involved with the control of lymphocyte activation are the co-stimulatory molecules on T lymphocyte surface. The interaction of CD28 molecule with B7 at the antigen presenting T cell is essential for lymphocyte activation, stimulating and sustaining cell proliferation. The increased B7/CD28 interaction is associated with exacerbated development of inflammation [33]. CD28 is stimulated in the first steps of T cell activation and it is related to IL-2 secretion, B cell proliferation and differentiation into antibody-producing plasma cells and intensification of proinflammatory gene expression by lymphocytes [34]. In the present study, we observed that PA reduced CD28 expression when compared to control or OA treated cells, at the both concentrations evaluated, suggesting that PA is able to inhibit lymphocyte proliferation by reducing the expression of CD28 and, consequently, cell activation. This effect may be related to the decrease of inflammatory cytokines IL6, IFN-γ and TNF-α promoted by this FA [34, 35]. In fact, CD28 binding to its ligands is associated to activation of transcription factors such as NF-AT, AP-1 and NF-κB leading to expression of inflammatory cytokine genes [36].

In contrast, expression of CD95 on the cell surface and its binding to Fas ligand (Fas L) are processes that initiate cell apoptosis [37], leading to an arrest of cell proliferation process. Some studies show that mutations in genes encoding CD95 may lead to the accumulation of peripheral lymphocyte, ultimately leading to the development of autoimmune diseases [38]. PA increased CD95 expression when compared to control and OA treated cells, indicating that this FA decreases lymphocyte proliferation through suppressing activation molecules and stimulating suppressor receptors, resulting in a reduced Th1 and Th17 response and inhibited inflammatory response.

The balance of inhibitory and stimulatory signals to the lymphocyte proliferation and activation determines the nature of the T cell response. Excessive lymphocyte activation can promote exacerbated inflammation and increase the risk for immune disease development [39]. The suppressive PA effect on lymphocyte proliferation ile may lead to a reduction in the production of inflammatory cytokines by Th17 and Th1, characterizing a possible anti-inflammatory effect of this FA. On the other hand, OA promoted stimulatory effects on lymphocyte activation maybe due to mechanisms involved with IL-2 production and Th2 activation.

## Conclusions

In conclusion, we found that PA has a suppressing effect on lymphocyte activation, promoting reduction in proliferative capacity. This effect is characterized by decreased production of inflammatory cytokines involved with Th1 and Th17A cell responses. Additionally, PA reduced the expression of activation molecule CD28 and increased the inhibitory receptor CD95, without involving Treg cell response. On the other hand, the stimulatory effect of OA on cell proliferation is related to IL-2 production, without interfering in other parameters. In summary, we found an anti-inflammatory action of PA on lymphocytes. Therefore, we believe that this research can contribute to the improvement of inflammatory immune disorders treatment, but more studies in relation to PA should be continued to achieve this goal.

## Abbreviations

ConA: Concanavalin A
FAs: Fatty acids
FBS: Bovine serum
IFN: interferon
IL: Interleukin
MUFA: Monounsaturated fatty acid
OA: Oleic acid
PA: Palmitoleic acid
Th: T helper cells
TNF: Tumor necrosis factor
